# Oral Prophylaxis

**Published:** 1908-07-15

**Authors:** Mabel M. Bennett

**Affiliations:** Detroit


### ORAL PROPHYLAXIS.

BY MABEL M. BENNETT, A.B., D.D.S., DETROIT.

Read before the Michigan First District Dental Society, April 9th, 1908.

Since taking up the work of Oral Prophylaxis, it has been a question in my mind of how the great "mass of people" are to be reached, and the benefit of treatment derived. I was fully convinced personally of its merits by visiting Dr. Smith's clinic in Philadelphia. The results of my own work, and the reading of such papers as Dr. Zederbaum's on "The Propagandism of Dental Education in the Public Schools," tends to increase my interest in the direction of the general public.

As the time allotted to me for the preparation of this paper has been so very short, and as Dr. Rogers in her paper on the "Treatment of Pyorrhoea" which most of you either heard or have read, admirably covered in detail the method of treatment, I shall not weary you tonight with a repetition of it. It is not the aim or purpose of the writer to bring before you anything original on the subject of the evening, but merely to present my personal opinions on a few matters, hoping they may serve as a means for a valuable and fruitful discussion.

How is this great work to be propagated? There are only a comparative few in the profession, general practitioners and specialists, who are securing beautiful and successful results, and it seems to me there is not the universal interest in it which should be manifested if the results desired by those most interested are to be obtained. Those of us who have not attended the "Torrey" services may have seen a good deal in the papers about a spiritual revival. I would recommend an Oral Prophylaxis revival for the profession of dentistry

We have all heard the saying, "Missionary work begins at home." I think it is very applicable in this case, for until the sympathy and interests of the members of the profession are enlisted in the work of oral prophylaxis and oral hygiene, little headway can be made with the laity. At best, it will be a slow, educational process, for this is a subject that has never been given the attention its importance demands. The chief aim of dentistry from its inception to the present time has been to repair the ravages of dental caries and restore lost parts. It is a subject of momentous importance both for the profession and the public. There is actual need of greater knowledge on the part of our professicn of the value of this form of dental service, the people need it, and respond gratefully to it when adequately administered.

What has a dentist to gain who is not in sympathy with this work? What object can he have for withholding from his patients prophylactic measures? It can be only a question of a short time when the public will force him to undertake it, or at least endorse it by referring his patients to those who do it, or he will be branded a back number. Oral prophylaxis is not a barren ideality but one of the most important and positive factors in the profession to-day. Patients, when asked how they lost a certain tooth, cast a reflection on their dentist by replying, "Oh, it was loose and Dr. So-and-so extracted it probably because he did not know how to save it."

The dentist who conscientiously attempts this treatment will find himself per force doing more skillful and painstaking work in every other department as he will detect for himself irritated conditions directly resultant from imperfect work. These observations will tend to make him perform subsequent operations with greater consideration for the soft tissues and raise his own work to a higher standard of excellency. It is, then, not only a primary benefit to the patient, butan equally desirableone to the dentist himself. He is compelled to recognize that the soft tissues are first in importance, and that oral prophylaxis should be given, not second, but first place in his practice, as it is fundamental and necessary to all success.

The majority of people are not adequately instructed regarding the care of their teeth and are totally unacquainted with inimical mouth conditions. If this be so, how then can they be expected to adopt any intelligent habit or system of treatment for the preservation of their teeth? There are people, to be sure, who cannot afford treatment regularly as the present professional specialist gives it, but are there any so poor, who, if properly instructed by a sufficiently interested dentist, cannot practice a simple habit of oral hygiene? The mere telling a patient about oral hygiene is not sufficient and I do not believe it ever will accomplish the desired results. Patients, as a rule, are not offended by the apparent unusual interest you take in presenting this matter to them. On the contrary, whenever an infectious condition of the mouth is made known to people of intelligence, and the relation of these conditions of decay of the teeth has been explained, they manifest the liveliest interest. It is the exception, rather than the rule, for patients not to cooperate in their efforts for improving threatening oral conditions. Patients will sometimes say, "I brush my teeth four or five times a day," but to look at them you would not think so; or, "I never had anyone show me how to brush my teeth; I did the best I could and thought I was doing it right." If it is an advantage to the adult to learn proper habits in the care of the teeth, can we estimate the advantage it should be to the growing child to form the proper habits for a life time?

This propaganda is a debt which we as dentists owe to humanity because of the influence it must have on the patient, whose estimate of his teeth will be no more than that which the dentist places upon them. It behooves the profession then to rescue and prevent as much as possible the loss of the teeth, if dentistry is to keep the place which it has occupied as a branch of the healing art.

The profession should be urged to be on the lookout for abnormal conditions and recognize pyorrhoea in its incipient stage. In order to detect this disease at an early period he must know what a perfectly healthy mouth looks like. These are rare, for no mouth is absolutely healthy which has not been put in such condition by treatment. The best way probably for one to find this out is to place one's self in the hands or a competent prophylaxis operator. The result will be so convincing that the most skeptical will realize the difference between a clean and an unhealthy mouth and when he takes up this line of work himself and sees the results he is certain to become an enthusiast.

In writing on oral prophylaxis reference is so often made to the preventive treatment, but so many cases require curative or rescue treatment before the preventive can be given that for the present our attention must necessarily be given to the curative that we may convince the profession of its practibility.

Since little is known regarding the pathology of pyorrhoea alveolaris, its treatment must to a certain extent be but groping in the dark, but whether the cause is local or constitutional matters little so long as one is able by technical means to cope with the disease. The method of treatment consists largely in the application of the oldest rule in medicine and surgery, the removal of the cause. The results will depend on the thoroughness with which the work is done, and anyone who attempts it with any other idea than that of painstaking thoroughness will be disappointed. Every vestige of hard or soft deposit must be removed and kept removed by the regular treatment of the dentist and personal care on the part of the patient. Some may ask what is the use of attempting to treat pyorrhoea at all? You cannot cure it. This question may be answered by asking another. Why fill teeth? You cannot cure caries. Teeth are filled to stop decay. Pyorrhoea on the same line of logic should be treated to stop the progress of the disease in order to prolong the usefulness of the teeth.

For the patient to do his part of the work, he should first be instructed in the intelligent brushing of the teeth; then equipped with at least two brushes, floss, and powder, appropriate for his use. For general cleaning, I think the "Rolling" brush (smaller adult size) is best. Cutters' dental floss is advised, either the standard size or the ribbon floss as the case requires. Nearly every mouth offers a different problem for solution in the matter of personal care, due largely to malocclusion produced either by faulty eruption of the permanent teeth or the drifting of the remaining teeth resultant from extraction. This prevents the teeth from having their proper exercise, invites inflammation of the gums, or caries. The average patient should be instructed to use the floss once a day, preferably at night, and to brush his teeth twice a day thoroughly.

There is probably no one for whose opinion the members of a family have so much respect medically as that of the family physician, and if dentistry is to succeed in its efforts for better oral conditions, we must get the medical profession interested. The family physician going into the homes, or even at his office, can emphasize the advantage of oral hygiene to the parents, who will see that greater care of their children's teeth is observed.

Oral prophylaxis is a common ground on which the physician and the dentist can meet. The medical profession may greatly aid if physicians can be made to recognize the dangers possible from a virulent mouth infection. The use of the dental mouth mirror by the physician who is trained to recognize an unhealthy or unsanitary mouth might be the means of discovering an obscure source of disease, as in tonsilar and laryngeal troubles, or even gastro-intestinal, or more remote systemic disorders.

The physician as well as the dentist owes it to himself to become acquainted with prophylaxis methods of treatment, that he may not only advise, but urge upon his patients the necessity of improved oral conditions. He, as well as the dentist, should recognize pyorrhoea in its incipiency.

In diagnosing a case how many physicians, when first called, think of the role the dental organs play in the patient at hand? Many physicians in treating a patient will be most particular about the surroundings in every other way than the mouth and let that remain in a filthy condition.

Have we not learned that the mouth is the avenue of infection of most specific diseases? If so, would it not seem reasonable that an effective way of combatting disease, would be to rid the oral cavity of its unhygienic, putrefactive condition? The swallowing of mouth accumulations containing micro-organisms and their products with the food disturbs not only the digestive tract but the whole system. To obtain the desired results by means of oral prophylaxis and to avoid many troubles under the jurisdiction of the medical profession, it is necessary to remove mechanically the media on which the germs of fermentation are growing. Observation has taught us that it is the fermenting and putrefying soft accumulations which act most viciously on the tissues. The hard deposit which accumulates on the teeth makes a roughened surface to which the soft irritating ones cling. The regular preventive treatment removes the bacterial plaques and other coatings from the teeth, stimulates the gums and peridental membrane and changes the whole environment of the teeth.

Dr. Horace Warren of Missouri Valley, Iowa, in speaking of the respects in which the micro-organisms of the mouth which produce decay and other pathological changes are similar to other parasites said, "Rats are not likely to hang around an empty corncrib nor mice hold conventions in deserted cubboards. So with the bacteria of the mouth. The impurities of the mouth form an excellent feeding ground. The temperature of the mouth 98.6 is just to their liking. Another factor is rest. That is the keynote to the prophylactic treatment of the mouth. The tramp who calls at your door does not ask for work. He wants rest. So with the bacteria of the mouth. They have been crying for this rest since man first trod the primeval forests, and if you allow them this rest which they desire, they will be doing business at the old stand till time shall end."

Since it is possible because of our knowledge of the habit and influence of micro-organisms to check the progress of bacterial development in the mouth, should not oral prophylaxis have a tendency to lessen the death rate and reduce the percentage of illness?

No one would dispute the assertion that it is impossible to have a perfectly healthy throat and lower air passages in association with an unhealthy nose; and yet there seems to be a surprisingly large number of physicians who see no incompatibility between diseased teeth and a normal digestive tract below them. It is my belief that there are many cases which the nose and throat, lung, and especially stomach specialists should refer to the dentist for treatment.

At our last meeting the matter of having a suitable textbook, to be used in public schools, on the hygiene of the body and particularly oral hygiene, to be edited by members of the medical and dental profession who have made extensive study of their respective subjects was discussed. It would seem that if members of health boards and school boards were alive to existing conditions and the possibilities that are obtainable through practicable improvement in mouth conditions, much could be accomplished from this source. In as much as it is a question of *health* conditions, the cooperation of these boards is indispensable.

Practical lectures on oral hygiene, by interested dentists, given occasionally at meetings of teachers whose cooperation would be needed in promulgating cleanliness of the mouth, and dentists as members of the school boards, dental clinics, etc., have been discussed to considerable extent and advocated by many as a wholesale and easy method of distributing practicable hygienic information.

While these means are all helpful, oral hygiene can only be accomplished by more direct means of education. Concerted action and earnestness on the part of the dental profession will be necessary to accomplish any radical change in existing conditions other than that which is bound to come by the old and slow process of gradual demonstration by a few earnest and diligent persons who are willing to "burn while they wait," for their own profession to wake up and realize that something is doing.

DISCUSSION.

Dr. Hoff. This is a most excellent presentation of this subject, in fact, I have never heard the real point at issue presented better than it has been here tonight. You have had quite a discussion here tonight in regard to raising money for use among the poor children in the schools. I do not want to throw any cold water upon this proposition, but I really do not believe you will accomplish your object even should you raise a thousand dollars, and to do the work I don't believe you can more than start it with the $300 you are asking. You raised a thousand dollars two years ago for a boat ride, and didn't have any trouble at all in raising it, and I don't see why you are so timid in asking for this small sum for so good a cause. Money, however, will not solve this problem as I see it. I think Dr. Bennett has presented to you the key to its solution, and if you will take it up in her way you will certainly make greater headway than you will in any other way. It is true, as Dr. Bennett has said, that there are only a few dentists who have experienced this change of heart, and have taken hold of this oral prophylaxis proposition in such a way that they have got "religion"—who have got the "oral prophylaxis religion," but these few are learning fast what can be done by means of oral prophylaxis. They are so enthusiastic about it that they stand much in danger of being called "cranks" on the subject. I must say that I am very likely myself to fall into this class, because I feel as though it is the best work that I have ever done in my professional career, and if I were to cut loose today from my moorings there is nothing that would catch me as soon as this prophylaxis idea. I should like to do nothing else but oral prophylaxis work, for it is fundamental, as Dr. Bennett has stated, and is certainly practicable. It is certainly the basis of all oral hygiene, and the necessary forerunner of preventive remedial measures. You may treat the teeth as much as you please; you may do as much as you can technically to restore the lost parts and lost functions of the teeth, but so long as you are allowing disease to spread itself, and so long as you are allowing infection to exhibit itself in the mouth, what progress are you making against this stream of destruction which is continually going on, growing and multiplying all the while. It seems to me that it is the part of wisdom to start at the fountain head; to begin with the prevention of the disease in the mouth. We can control dental caries, and we can control disease of the gums; we can control other diseases of the mouth, to say nothing of the general systemic conditions which are influenced many times because of diseased conditions in the mouth. We can prevent these diseases by oral hygienic means, and this system of oral prophylaxis is the nearest approach to a genuine treatment, or a genuine application of oral hygienic means, that I have ever known anything about, and I wish to add my personal testimony in just as emphatic a way as I know how to this audience. This treatment, as we are practicing it today, is the most perfect hygienic method that I have ever had anything to do with, and I believe all who have practiced oral prophylaxis faithfully and conscientiously for any length of time will give the same testimony. I have yet to find anyone who has done this work, conscientiously and intelligently who has given any other kind of testimony to me. I believe most heartily and cordially that Dr. Bennett has struck the keynote in another direction also, which is this, that our profession is asleep on this subject. We are not wide awake and making ourselves intelligent on this subject as we should be. It is not a difficult matter to master and no one can take up this work and do it conscientiously, even for a short time, without seeing satisfactory results and being convinced that there is more to it than he ever dreamed of before. If you cannot devote yourself to this work as a specialist, you should do what you can as it comes your way, and in doing so you will awaken a new interest in the cleanliness in the mouth in your own practice and among your patients that will have a far wider influence upon the public generally in the matter of oral hygiene than all the school work that you could possibly do. If every one of you would give a day out of every week to treat children in the public schools, all you will do there will be to relieve the toothache, or fill a few teeth with amalgam and cement, and you will not be doing educational work at all. You will simply call the attention of some of them to the fact that they have decayed teeth that ought to be filled, and when you fill these teeth you will hurt them so much that they will dread the sight of a dentist, and all your work will be lost. We ought not to waste our strength and energy in that kind of work, because it is only transitory in its results; it leads to nothing. What we ought to do is to build up this idea of oral hygiene and cleanliness in the mouths of our own patients, and through our patients let us spread this good news to others. To me this is not a dream at all, nor an idle notion, because I know what I have been able to do in my own practice. I have been able to introduce to my patients a different set of hygienic conditions than ever have prevailed there before. And what I have done for them has spread from my own patients to other people who have heard about this work and are inquiring about it, and wanting to know what it all means. If we could get all our patients enthusiastic over oral prophylaxis they would gladly spread the good news, and when the time comes when money is needed for propagating public hygiene you will not need to ask each other for money to propagate this thing for the poor people, because your own patients will be glad and willing to give money to help on this cause among the worthy poor. One of the strongest points made in Dr. Bennett's paper is that the dental profession must be enthused on this subject before we can make any progress at all in general hygiene. We do not need to specialize in this work to become enthusiastic, but we ought to know more about it, and practice it when we find opportunity. Another valuable point made by the essayist was, enlisting the aid of the medical profession. I would suggest that every member of this society who has a physician among his clientele, he should put his mouth in a thoroughly hygienic condition. There is not a physician in Detroit but needs this work—some of them seriously. Get them into your chair as soon as you become proficient enough in the work to make a proper impression on them, and you will convert them, and through them you will do an enormous amount of good. All such physicians then will wake up to the benefits of dentistry as a factor in general hygiene. I believe through wakening up our own profession, and allying with us the help of the medical profession, we can do a great deal more good than can possibly come from any propaganda that you might be able to establish with money in the way of taking care of conditions already existing in the mouths of poor people, or young people in our public schools, or by any system of public charities or clinics.

Dr. C. P. Wood: I hesitate to say anything after this very excellent paper, and after what has been said by Dr. Hoff. The paper certainly could not be improved upon at this day and date. I was not surprised, because I have seen work done by the essayist, as good work as I have seen in the line of oral prophylaxis treatment. Dr. Bennett says that she received her first important stimulus from Dr. Smith. There are many of us who can say the same thing; he gave me such a boost that I have not got over it yet, he set my heart throbbing so that it is going so fast now that I cannot speak because of enthusiasm for the subject of oral prophylaxis. I advocate it more now for the curing of disease than I ever did before. It has grown on me for several years, and if I had to give it up to-day I would quit dentistry absolutely;—I could not believe I was accomplishing anything. When the physician or dentist can keep to the preventive treatment in his practice, he is making the highest success in his profession. How much better it is for a physician to prevent all sorts of diseases, the same as they are in the public schools, in stopping diphtheria and all those things, just by the preventive methods, and so with the stopping of pyorrhea, as we have demonstrated it, some of us who have pushed it to a successful issue, that pyorrhea will not exist and cannot exist in a mouth where prophylaxis is thoroughly practiced. Prophylaxis has been practiced by so many that it is not so new that we need be afraid of it. There are several men in the country who have practiced oral prophylaxis for at least twenty-five years. I believe Dr. Smith has put it on a more practicable basis for the profession, more particularly for the last ten years. He has proven it in his own practice to be an absolute success, and claims he can stop from 75 to 90 percent of dental caries by oral prophylaxis treatment alone. I cannot do that yet, but I believe he can. It is very much of an evolutionary practice; we learn something new from it every day, and that is the beauty of it. It just keeps us on the top notch all the time, and makes us enthusiastic over every part of our work. To give an illustration, I formerly would put on a crown that when I attempted to polish the teeth I would bump up against its edge, but now I put on that crown so that you cannot strike the edge at all; you slip over that; it is going to be pretty nearly a flush joint. You realize the necessity of a perfect fit of all crowns.

Speaking of prophylaxis being a new thing. I have a physician one of whose patients is taking prophylaxis treatment in my office. This patient came to me, and a prettier mouth I never looked into. He had not been in a dentist's office for five years, but a good dentist had put gold fillings in his teeth 25 years ago, and this dentist gave him a few prophylaxis lectures, and this man says: "I have brushed my teeth five times a day for twenty years." That dentist actually started the prophylaxis treatment as much as any of us, I guess. I tell my patients, and insist on it, that they should brush their teeth before breakfast, five to ten minutes, as the case requires, to get the mouth clean; also before retiring and after each meal. This patient brushed his teeth five times a day for twenty years, and they were not worn out yet. The surfaces were polished, and his gums were as hard as could be. That proves to me that some cases, if not all cases, need to go to the dentist once a month. There are erroneous ideas along this line, that every patient needs to go to the dentist once a month; I do not think that is so at all. I instruct my patients, and get them to take such such good care of their mouth that they keep the tissues in good condition, so that all do not need to come to me once a month. Then I say, "You come once in two months now" and, when they get still better, "Come once in three months," and so on.

I think our most important work in prophylaxis is to instruct the patients. A man was in my office to-day from out in the state, and I had treated him for a severe case of pyorrhea and he needed to come to me for a few months. I made him come twice a month, that I might instruct him in taking care of his own mouth. I would instruct him as to this place, or that place in his mouth, from time to time, if he did not get it in perfect order, and after talking with him a few times, and doing some little work for him in polishing the teeth, with the usual operation of polishing and the floss, etc., I said, now, Mr. —— it depends mostly on you how this work comes out. It is now clean and splendid, whereas two or three months ago it was terrible—teeth loose all around. Very much now depends on how you care for your teeth, I say to them. I also say to them, I will tell you from time to time if you are not properly caring for them, and by and by you will be able to take care of them yourself to the best advantage. I speak of this because I believe it is important. We gradually grow into these things and find the importance of them as we go along, if we can pick them out without waiting or stumbling onto them, so much the better. There are a few dentists in this city who tell their patients that they should not take prophylaxis treatment, that it is a wrong idea, and that it will wear out their teeth, and be harmful in many ways. I think that is the most outrageous advice that ever could be given by any dentist. It seems to me that it is pure jealousy or laziness. A patient goes to such an office, and the dentist will put his mouth in good condition, and then tell him he don't need prophylaxis treatment, but to come back after a year or two, or when he feels like it. I think such a dentist is doing his patient absolute injury, because there is not one mouth in a thousand but will come back in bad shape in even a year. Even very poor work will be tolerated in a mouth that has regular prophylaxis treatment; I think all who practice it have noticed this fact, especially where a crown or bridge would be likely to produce unsanitary conditions if left to the patient entirely without the prophylaxis treatment. Such mouths keep in fair condition, even with bad work, providing it can be kept clean by the dentist.

Dr. Smith has said that there could not be pyorrhea in a mouth where prophylaxis is practiced, and I believe it absolutely.

My patients are much pleased with the treatment and they are willing to pay me reasonable fees for the time. We must not however, get the idea, that every patient who comes to us can pay four or five dollars an hour for this treatment. I have many patients, whom I took on in my first year of pushing this prophylaxis, whom I put on regular sittings for one-half of that rate. And while I believe it is right for a dentist to charge so much an hour for his work, for the sake of experimenting or of educating the patients to take care of their mouths, I am willing to just put on some of my time with some patients for even a nominal fee. I believe that it is so important that if the patients cannot pay three, four or five dollars an hour, as the case may be, they should get the service for less, if they need it.

Dr. Hoff: In regard to criticism that dentists make about prophylaxis, I recently heard an eminent dentist make the statement that prophylaxis work did more harm than good, and that he had seen cases where the gums had been almost ruined by practicing prophylaxis. I asked him if he had ever done oral prophylaxis himself, and he said no. I asked him about the case to which he referred and he said that it was a case done by a student in a college clinic. That is the kind of criticism you will hear about this work, and it is worthless because without knowledge.

Dr. E. B. Spalding. I can most heartily say "Amen" to this paper. Dr. Bennett, in her paper, asked the question: "What is the dentist going to gain by not advocating prophylaxis?" He does not think he is going to gain anything, because he does not know anything about the result, if he does not advocate or practice it, he has not been converted. The matter that Dr. Wood has mentioned is something that should not be lost sight of. In the first place most dentists will not work if they are not going to be suitably remunerated and unless we do conscientious work the patient will not have the full benefit. Dr. Bennett said the patient will not value the teeth any more than their dentists do, and if the dentists do not value their operations and their skill and time enough to exact a suitable fee either they are not producing results or have very biased ideas of the work and will not do it properly, and if the patient does not leave a suitable fee for it, he likewise will not value it as he should. There is no harder work in dentistry than this prophylaxis work or the treatment of pyorrhea. I advocate a suitable fee to be paid for this work, and I think the dentists will then begin to realize that it is of some importance in a financial way.

Dr. Wood: I think that statement of mine needs a little more said about it. What man is there in this room who gets so many dollars an hour for a gold filling, an amalgam filling, gold inlays, porcelain inlays, for this patient and that patient, week in and week out? I venture to say there are not five men in the room who get the same fee from every patient. A very reputable dentist stated to Dr. Thompson, about twelve or fifteen years ago, that he charged some persons $12 for a Logan crown and other persons $5 or $7, or whatever it might be, for the same operation, and I said then that that dentist was not doing right. After twelve years however, I have grown to know better, and if I have a patient who is able to pay me $15 or $20 for a crown I am going to get that, and he will get value received. If I have another patient who cannot pay more than half as much, I am going to do that patient good work, if I know how, and charge him as much as he can pay. Now I would not say that to every patient who came into the office; if you do you will finally have nothing but the upper crust—which is nice, but every man cannot have it.

If you are going to do missionary work you cannot always get $5 an hour for it; you may have to put in $5 an hour to get the people to have it. I have lost $5 an hour to get patients to have prophylaxis when they did not know they needed it. A man who absolutely cannot afford to pay more than $2 a month for treatments, why should not that man get that treatment, and conscientiously, too. I have had a pyorrhoea patient, in my practice which I could not control with monthly treatments. I had him come every two weeks for a straight year and charged him accordingly and I finally cured the case and got it where it is now in perfect condition. If we do justice to the case many times we must not always hold to a set fee.

Dr. Burke: Dr. Bennett's paper this evening should prove a great stimulus to the Committee that has been appointed for the purpose of bringing about oral hygiene in the public schools. Personally, I regret that Dr. Hoff has discouraged the work of that Committee by his remarks. I believe that the question of examining the mouths and teeth of the children in the public schools is a more important question than that of general oral prophylaxis. My reason for saying that is just this. We cannot always rely upon statistics, but I have read in the work of an authority on this subject that about 25 percent of the people never visit a dental office, and I have also read in the dental journals that only about ten per cent of the children in the public schools clean their teeth with a suitable brush and powder. Now if our committee is ultimately successful in bringing about these examinations in the city of Detroit, you can readily see that when this work is put into operation and the little children are sent to their family dentist once or twice a year, that you are going to reach a much larger percentage of the people than the progressive men in our profession interest through prophylaxis. The important point at this time is to bring about a state of affairs whereby we can induce the children, particularly in the public schools, to visit the dentist. That, it seems to me, is a very important thing. Now I would suggest that before any effort is made to collect money from the men in this city who are interested in this work, that a copy of Dr. Bennett's paper be mailed to the members of the profession in this city, so as to start them thinking along this line. It seems to me that it would make it a great deal easier for the committee which the President is going to name this evening. I believe we all ought to interest ourselves with this committee in trying to bring about a thorough examination of the mouths and teeth of all the children in our public schools. If our committee is successful in this work that both the public and the profession will be greatly benefited.

Dr. D. M. Graham: I want to compliment the essayist on her paper. I think it a valuable and instructive paper and, considering that she had so few weeks for preparing it I think it is all the more credit that she should present us so valuable a contribution. I am not so enthusiastic as some of the gentlemen who have spoken as well as some other writers on oral prophylaxis and hygiene, for the very good reason that possibly I do not do so much of it as they do. I know it is capable of great things, and believe that if employed soon enough and often enough that it will prevent pyorrhoea. I believe also that if employed often enough and skillfully enough it will cure pyorrhoea in a great majority of cases. The status of oral prophylaxis in regard to the prevention and cure of pyorrhoea I believe is fairly well established. Its status in regard to the prevention of decay I believe is not so well established. I believe it would be well for our prophylactic enthusiasts to gather some statistics along this line and inform us what, in their opinion, is the status of oral prophylaxis in regard to the prevention of caries. We have all heard of Dr. Smith, and a few others, saying that it is capable of preventing 90 per cent of caries. While I am not in a position to successfully contradict that, I do not believe it is capable of doing it, and were it capable of preventing caries to the extent of 10 per cent I believe it would behoove us to employ it in our practice more than we do. It has often been a matter of interest to me, if oral prophylaxis is such a preventive remedy locally as well as systemically, why the gastrologist and the specialist on nose and throat, should give it so little attention. I know at least half a dozen men in this city who are doing special work along the lines of rhinology and I have asked them, each, this question: "What, in your opinion, is the value of oral prophylaxis in preventing and curing diseased conditions of the air passages?" I was not entirely surprised that their answer was that they did not know that it was of very great aid. The trouble with them is, as it is with the dentist, that they do not understand the value of it themselves. Physicians are not educated to the advantages of oral hygiene and oral prophylaxis. They will tell you that they do not see any less tonsillar inflammation, pharyngitis, laryngitis among those treated regularly by our prophylactic enthusiasts than they do with those who are not; but the work has not been carried on long enough to draw any particular conclusions. If that be true it would seem to me that it is going to assure us in affirming or contradicting much of what has been said. At one time I became quite an enthusiast and thought that by the employment of oral hygiene and oral prophylaxis, gastritis, enteritis, and tonsilitis and several other "itis" could be cured, or possibly be prevented in many cases. I attempted in a few cases that line of treatment. I was not entirely successful, and because I was not I am not prepared to discourage oral prophylaxis, but I do think that too much is claimed for it in the way of prevention in regard to systemic infection. While much good can be had and much prevention is undoubtedly obtained by just such treatments, I do not think that it will prevent appendicitis, cancer, and inflammation of the gall-bladder, and all that, as has been said by some of our enthusiasts. I sometimes think that, while enthusiasm is necessary to carry a new subject to a successful issue, that there is also a great deal of harm in being too enthusiastic and sanguine and radical. I believe there are none of us who have seen this work who doubt for a minute that prophylaxis is the only treatment, when applied to pyorrhoea, but I think a good many of us have our doubts as to the extent of its usefulness in preventing caries, and also what its function in regard to systemic infection is.

Dr. Spalding: I am very glad that Dr. Graham spoke as he did, because it is honest. He won't let his enthusiasm run away with him. I do not believe that there is any way possible to estimate the exact percentage of good that has been done, but there are two or three things you can think about. One is that Dr. Black took a patient, that was an immune, and had no caries whatever, and by putting upon a molar a band which had a shoulder near the gum, in three months time he produced a very large surface of decay, where the accumulations had gathered. It was thoroughly cemented, as I understand it. It would seem as if the conditions were suitable in any mouth, caries would result, and that the lack of a smooth surface, and even smooth surfaces which accumulate soft deposits will produce decay. As to his saying that he did not know how far it was a prevention of some of the laryngitis and tonsilitis, we have a patient who is a prominent singer in the city, who was troubled for two years with a great deal of sore throat and hoarseness, and his mouth was far from a healthy condition; there was a great deal of inflammation of the soft tissues and heavy deposits. He had not been under treatment more than three months before he began to improve, and in six months was in fine condition, and today he is one of our most ardent supporters of prophylaxis. His trouble was of long standing before he took up the treatment. How far the prophylaxis treatments were responsible in curing this case I cannot say certainly, but it seems as if it must have had some effect.

Dr. G. P. Rogers: I feel as if I ought to say something in behalf of the committee appointed on oral hygiene. Dr. Hoff does not believe we will accomplish anything if we do get the money, and Dr. Burke seems to think that there is doubt. Now we want you to know that we are going to accomplish something, and we believe we are going to be supported by the Society. Of course it is now we need the money, and we know that we are going to get it. Dr. Hoff also suggested that all we did would be to fill a few teeth that were aching, but that is not all we are going to do. We will precede our operative work by a campaign of education. We hope this spring to have some one address the mothers meetings in the vicinity of the few schools that we will work in, and we hope to give talks to the children before they receive any examination at all, and we are sure that we are going to accomplish something and want your support.

Dr. Straith: I want to endorse what Dr. Rogers has said. This is a matter of education. It was my good fortune to be reared and brought up among rural people, and I know from my personal experience that oral prophylaxis does much more than we give it credit for. Fourteen years ago when I entered college I had not seen fit to take advantage of the good advice given me in the way of taking care of my teeth, and I lost many of them since I entered college. Ninetenths of our teaching is upon the preservation of the teeth; the other tenth is incidentally to replace that which has been lost, with something that never can compare with the original in any state. I knew that when that was taught to me and I started out to tell it to other people, I must do some of it for myself. I began telling them that which was taught me, and by that time had a number of my teeth that were soon ready for the waste-basket, fixed up, and I have got all those yet, and have not lost any more, and I think that with a little care I do not know any reason why I should ever lose any teeth. I was reared in a rural community and it was my privilege to begin practice in a rural community. I practiced ten years in a little town where my predecessor had taught the people very little except extraction and putting in plates. Notwithstanding my present specialty of extracting teeth only, I pat myself on the back and tell myself that I do not think there is another man who has used more of his powers of persuasion to teach people to save their teeth than I have. I am conscientious in that, and I know that there are men here tonight who know that I have after seeing patients, refused to extract their teeth, and sent them back to their dentists to have the teeth filled and cared for. I know when I began practice in the little town (and I began after my first year in college my first summer in practice) I was privileged to work for a young lady, as I remember, less than 20 years of age, whose mother was wearing full upper and lower dentures, whose parents knew nothing of the care of the teeth except their extraction and the wearing of plates. I did something like seven or eight dollars worth of work, wholly amalgam fillings, and charged $1 for one of them and 75 cents for another, and 50 cents for the rest of the fillings. If I ever got a calling down for myself it was from that mother because I had sent them such a bill, and not one gold filling. She said, "I could have had a gold plate made for that price." That was the class of people I had. However, I had the privilege and pleasure of having the younger members of that family come to me every two or three months before I left that little town, to have their teeth examined and filled. I know, from my experience among the rural people, that it is a matter of education with them, and while some of us who reach the better class of people who need very little instruction on that question can tell their people to take care of their teeth, brush them and have them scaled or taken care of ever so often, it goes all right with those people, but they do not associate with the class of people that this committee is trying to reach. They will reach a class that cannot be reached by the average dentist that is in this meeting here tonight. I would like to add what emphasis I could to the work which has been started, and, while I never was less prepared than I am now to aid this work in the way of money, yet I am here with the goods.

Dr. Bennett: Another point I wished to speak of is that I most heartily approve of the work of this committee. I am afraid that some of you have inferred that I did not approve of dentists introducing prophylaxis who have not practiced it. What I wished to say is that the dentist who has not practiced oral prophylaxis ought not to use his influence in opposing it. I also wish to thank the members of this society for their expression of appreciation of my efforts.

